# Bioresorbable vascular scaffolds for percutaneous treatment of chronic total coronary occlusions: a meta-analysis

**DOI:** 10.1186/s12872-019-1042-2

**Published:** 2019-03-15

**Authors:** Alberto Polimeni, Remzi Anadol, Thomas Münzel, Martin Geyer, Salvatore De Rosa, Ciro Indolfi, Tommaso Gori

**Affiliations:** 1 Kardiologie I, Zentrum für Kardiologie, University Medical Center Mainz and DZHK Standort Rhein-Main, Mainz, Germany; 2 Division of Cardiology, Department of Medical and Surgical Sciences, “Magna Graecia” University, 88100 Catanzaro, Italy; 3 URT-CNR, Department of Medicine, Consiglio Nazionale delle Ricerche of IFC, Viale Europa S/N, 88100 Catanzaro, Italy

**Keywords:** Bioresorbable vascular scaffold, Chronic total occlusion, Meta-analysis

## Abstract

**Background:**

BRS represent a new approach to treating coronary artery disease. Beneficial properties of BRS regarding the restoration of vasomotility after resorption make them attractive devices in CTO revascularization. However, experience in this setting is limited.

**Methods:**

We systematically searched Medline, Scholar, and Scopus for reports of at least 9 patients with CTO undergoing BRS implantation. Patients’ and procedural characteristics were summarized. The primary outcome of interest was target lesion revascularization (TLR). Pooled estimates were calculated using a random-effects meta-analysis. The study protocol was registered in PROSPERO (CRD42017069322).

**Results:**

Thirteen reports for a total of 843 lesions with a median follow-up of 12 months (IQR 6–12) were included in the analysis. At short-term, the summary estimate rate of TLR was 2.6% (95% CI: 1 to 4%, I^2^ = 0%, *P* = 0.887) while at mid to long-term it was 3.8% (95% CI: 2 to 6%, I^2^ = 0%, *P* = 0.803). At long-term follow-up (≥12 months), the summary estimate rate of cardiac death was 1.1% (95% CI: 0 to 2%, I^2^ = 0%, P = 0.887). The summary estimate rates of scaffold thrombosis and clinical restenosis were respectively 0.9% (95% CI: 0 to 2%, I^2^ = 0%, *P* = 0.919) and 1.8% (95% CI: 0 to 4%, I^2^ = 0%, *P* = 0.448). Finally, the summary estimate rate of target vessel revascularization was 6.6% (95% CI: 0 to 11%, I^2^ = 0%, *P* = 0.04).

**Conclusions:**

Implantation of BRS in a population with CTO is feasible, although further longer-term outcome studies are necessary.

**Electronic supplementary material:**

The online version of this article (10.1186/s12872-019-1042-2) contains supplementary material, which is available to authorized users.

## Background

Chronic total occlusions (CTO) are present in about 20% of patients with coronary artery disease undergoing elective angiography [1]. Nevertheless, these lesions represent only a minority of the lesions treated with percutaneous coronary intervention (PCI), even if their treatment is associated with better outcome in terms of angina relief, improved left ventricular function, reduction in the rate of myocardial infarction and coronary artery bypass grafting (CABG), and potentially prolonged survival, particularly in the setting of multivessel disease when complete revascularization is achieved [2].

After successful recanalization of the vessel, stenting is mandatory, preferably with drug-eluting stents (DES), to ensure long-term vessel patency [3]. Although favorable long-term outcome data have been reported after the implantation of DES, the implantation of multiple metallic stents into coronary arteries may lead to an augmented risk of restenosis and thrombosis, impairment of vasomotion and positive remodeling and excludes the possibility of future bypass graft anastomosis within these segments [4]. In this setting, bioresorbable scaffolds (BRS) might therefore have potential advantages: avoidance of long coronary segments covered with metallic prostheses, restoration of endothelial function and normal vasomotor tone at least within noncalcified segments, long-term favourable vessel remodeling; finally, struts resorption preserves the possibility of further interventions by percutaneous or surgical means [5].

Conversely, there are also many limitations of BRS use in this subset of lesions: severely calcified vessels may be poorly accessible for bulky devices, and their low radial strength bear the risk of vessel recoil and underestimation of vessel size raise the risk of malapposition.

Importantly, CTO lesions were excluded in all BRS randomized controlled trials published to date [6–9], and all available evidence derives from small single-center, single-arm studies. We therefore undertook a systematic literature review and meta-analysis of studies examining the clinical outcomes of patients with chronic coronary occlusion undergoing BRS implantation.

## Methods

### Search strategy

Electronic searches were performed using Pubmed, Scholar, and Scopus electronic database up to June 13th, 2017. We checked the reference lists from all eligible studies to identify additional citations. The following keywords and the corresponding MeSH terms were used for search: “bioresorbable vascular scaffold”, “chronic total occlusion”, “coronary artery disease”. Time of publication was not limiting criterium for our analysis. All reports including the search terms were independently screened by two investigators for relevance and eligibility (AP, SDR) and any disagreement was resolved by consensus. The study protocol was registered in PROSPERO (CRD42017069322).

### Study selection

*Inclusion criteria*: 1) patients with at least one coronary chronic total occlusion 2) reports of a minimum of 9 patients with a follow-up at least of 1 month; 3) original articles reporting at least one of these outcomes: target lesion revascularization (TLR), target vessel revascularization (TVR), scaffold thrombosis (ScT), scaffold restenosis (ScR), cardiac death and 4) reports written in English language.

*Exclusion criteria*: 1) duplicate publication 2) pre-specified endpoint 3) measure not specified. If duplicate studies were identified, only the most exhaustive and recent reports were retained.

### Data extraction

Baseline characteristics as well as numbers of events were extracted from the single studies, through scanning of the full article by two independent reviewers (AP, SDR). Divergences were resolved by consensus.

The following data were abstracted: year of publication, location, number of study patients, study design, clinical outcome data, baseline patients’ characteristics, and procedural characteristics.

### Study endpoints

TLR was the primary outcome of interest. Secondary outcomes were TVR, ScT, clinical ScR, cardiac death.

### Statistical analysis

Categorical variables are reported as numbers and percentage, and continuous variables are reported as mean ± SD or median ± IQR. Random effects meta-analysis was conducted in all analyses using the Metaprop command, which allows computation of 95% confidence intervals (CIs) using the score statistic and the exact binomial method and incorporates the Freeman-Tukey double arcsine transformation of proportions [10]. Heterogeneity among studies was assessed with the I^2^ statistic. The effect of study-level covariates on the rate of TLR, ScT and ScR was explored with a meta-regression analysis by using the metareg command (Additional file 1). All analyses were performed with OpenMetaAnalyst software version 0.15 [11] and Stata statistical software version 13 (StataCorp LP, College Station, Texas).

## Results

### Search results

**Fig. 1:**
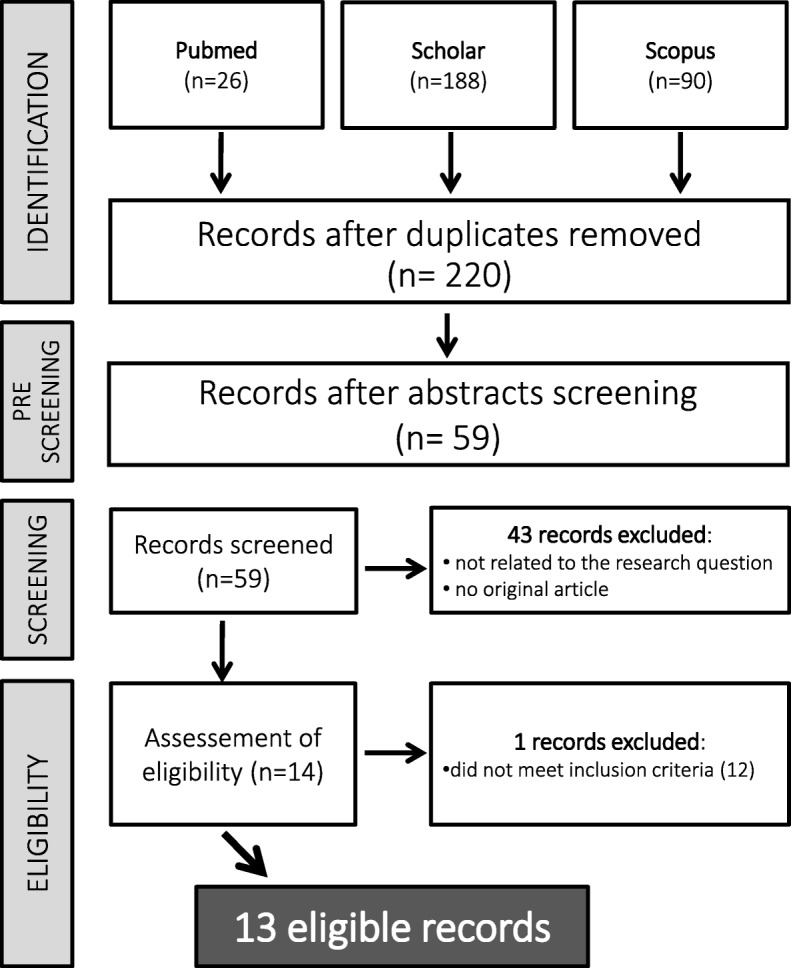
Study selection flow chart

Our search retrieved a total of 304 entries, which were reduced to 59 studies after an initial pre-screening. 43 studies were then excluded for one of the following reasons: a) they were not related to our research question b) they weren’t original articles. In the assessment of eligibility 1 additional study was excluded because as it is limited to in-hospital outcomes [12]. Finally, a total of 13 studies [13–25] with a median follow-up of 12 months (IQR 6–12) were available for the analysis including 843 lesions. The study selection procedure is reported in detail in Fig. 1.

### Study characteristics

Table 1 summarizes the patients´ most relevant baseline characteristics for each study.

**Table 1:** Baseline patient’s characteristics

	Abellas et al. 2017	Azzalini et al. 2016	Fam et al. 2017	Goktekin et al. 2015	Kugler et al. 2017	Lesiak et al. 2016	Mitomo et al. 2016	Ojeda et al. 2015	Ozel et al. 2016	Saad et al. 2016	Vaquerizo et al. 2016	Wiebe et al. 2015	Yamac et al. 2017
Age (years)	59.2 ± 8.7	60.0 ± 9.3	59.40 ± 8.96	56.9 ± 9.4	60.5 ± 7.8	59.9 ± 8.3	60.8 ± 11.0	58 ± 9	61.9 ± 9.7	65.3 ± 10.9	61 ± 10	60.4 ± 9.0	57.8 ± 9.6
Male (%)	–	89.5	89.5	90.0	85.7	77.5	89.2	98	85.4	75	80	81.8	86.7
Hypertension (%)	44.4	65.4	69.5	78.6	64.3	80	67.8	57	80.5	78.6	–	91.3	80
Diabetes (%)	22.2	34.0	33.3	21.4	14.3	30	40	33	51.2	26.3	20	34.8	3.3
Smoking (%)	77.8	24.8	48.6	35.7	57.1	35	–	8	34.1	41.5	–	47.8	40
Family History (%)	–	29.6	21.9	32.9	–	–	–	–	–	30.8	–	–	33.3
Hyperlipidemia (%)	100	69.9	72.4	52.9	71.4	–	61.5	64	46.3	50.4	–	65.2	56.7
Prior CABG (%)	–	2.6	2.9	10	0	5	6.2	–	17.1	2.7	–	–	6.7
Prior PCI (%)	–	43.8	46.7	17.1	–	45	53.8	36	56.1	39.3	–	–	13.3
Prior stroke/TIA (%)	0	2.6	–	0	–	–	30.8	–	–	–	–	–	0
CKD (%)	–	5.5	–	2	7.1	15	40.1	–	0	–	–	–	0
LVEF (%)	–	53.2 ± 10.1	–	51.7 ± 6.7	–	50.7 ± 10.2	57.7 ± 10.8	54 ± 8	–	59.8 ± 13.8	–	55.7 ± 15.5	50.2 ± 6.4

Across studies, patients were predominantly male and had a mean left ventricle ejection more than 50% while the percentages of patients with diabetes (3.3–51.2%), smoking (8–77.8%) and prior-PCI were variable (13.3–56.1%).

Lesion and procedural details are provided in Table 2. The percentage of lesion with moderate/severe calcification (0–70.5%) and that of lesions with a J-CTO score more or equal than 2 (26–100%) were variable while the percentage of post-dilation was almost similar and more than 69.6% in all the studies with the exception of the study by Saad et al. 2016 (25.7%).

**Table 2:** Lesion and procedural characteristics

	Abellas et al. 2017	Azzalini et al. 2016	Fam et al. 2017	Goktekin et al. 2015	Kugler et al. 2017	Lesiak et al. 2016	Mitomo et al. 2016	Ojeda et al. 2015	Ozel et al. 2016	Saad et al. 2016	Vaquerizo et al. 2016	Wiebe et al. 2015	Yamac et al. 2017
LAD (%)	22.2	46.4	41.9	51.4	20	57.5	46.2	48	34.1	41.6	–	43.5	34.3
LCX (%)	0	19.0	12.4	24.3	6.7	7.5	12.3	24	17	26.6	–	8.7	25.7
RCA (%)	77.8	34.6	44.8	32.9	73.3	35	40	28	48.7	31.7	46	47.8	40
Moderate/Severe calcifications (%)	66.7	45.8	70.5	28	46.7	30	32.3	–	–	0	34	65.2	22.9
J-CTO score ≥ 2	55.5	42.5	100	–	60	55	64.6	46	29.2	–	26	–	–
RVD (mm)	3.39 ± 0.22	3.0 ± 0.4	2.71 ± 0.55	–	3.24 ± 0.46	2.48 ± 0.33	2.97 ± 0.36	3.03 ± 0.4	2.8 ± 0.25	3.1 ± 0.5	2.48 ± 0.48	–	3.02 ± 0.39
Mean number of BRS implanted	3.22	2.2 ± 1.1	2.44 ± 1.12	2.01 ± 1.0	3.2 ± 1.3	1.6 (1–4)	1.8 ± 0.7	2.6 ± 1.9	1.27	1.63	–	2.8 ± 1.0	2.3 ± 0.9
Mean BRS diameter (mm)	3.29 ± 0.31	3.2 ± 0.4	3.00 ± 0.31	3.0 ± 0.4	–	2.90 ± 0.32	3.0 ± 0.4	3.030 ± 38	2.8 ± 0.29	3.1 ± 0.4	–	3.1 ± 0.2	3.2 ± 0.4
Total BRS length (mm)	21.93 ± 6.45	51.3 ± 24.1	59.75 ± 25.85	36.5 ± 19.5	81.7 ± 29.1	42.4 ± 21.5	47.6 ± 19.9	43 ± 21	25.6 ± 4.2	26 ± 14.7	53 ± 23	64.8 ± 24.2	58.3 ± 23.3
Post-dilation (%)	88.9	90.8	89.5	100	100	95	100	100	97.5	25.7	63	69.6	100
Mean post-dilation Balloon diameter (mm)	3.45 ± 0.28	3.3 ± 0.4	3.35 ± 0.44	3.5 ± 0.4	3.3 ± 0.4	3.15 ± 0.35	3.3 ± 0.3	–	–	–	–	–	3.4 ± 0.4

### Meta-analysis

**Fig. 2:**
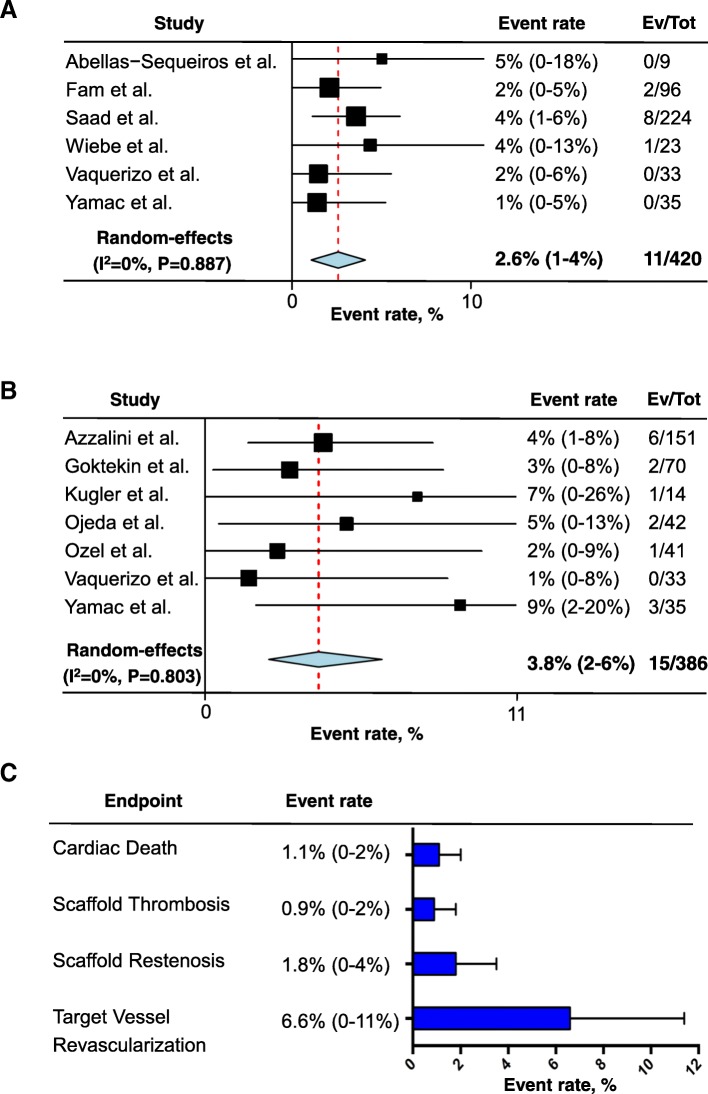
Random effects meta-analysis of target lesion revascularization (TLR) at short-term (panel **a**) and mid to long-term (panel **b**) follow-up. (panel **c**) Random effects meta-analyses of cardiac death, target vessel revascularization, scaffold thrombosis and restenosis at mid to long-term follow-up

The primary analysis on the composite endpoint of TLR both at short- (< 6 months) and mid to long- (> 11 months) term follow-up including all results of the studies is presented in Fig. 2. At short-term, the summary estimate rate of TLR was 2.6% (95% CI: 1 to 4%, I^2^ = 0%, *P* = 0.887, Fig. 2a) while at mid to long-term was 3.8% (95% CI: 2 to 6%, I^2^ = 0%, *P* = 0.803, Fig. 2b).

Secondary endpoints are reported in Fig. 2c. At mid to long-term follow-up, the summary estimate rate of cardiac death was 1.1% (95% CI: 0 to 2%, I^2^ = 0%, P = 0.887, Fig. 2c, first row). The summary estimate rates of scaffold thrombosis and clinical restenosis were respectively 0.9% (95% CI: 0 to 2%, I^2^ = 0%, *P* = 0.919, Fig. 2c, second row) and 1.8% (95% CI: 0 to 4%, I^2^ = 0%, *P* = 0.448, Fig. 2c, third row). Finally, the summary estimate rate of target vessel revascularization was 6.6% (95% CI: 0 to 11%, I^2^ = 0%, *P* = 0.04, Fig. 2c, fourth row).

### Meta-regression analysis

Given the differences between Japan-Chronic Total Occlusion (J-CTO) score between the studies, we used the percentage of interventional procedures with J-CTO ≥ 2 in every single study as a moderator in a meta-regression analysis with the effect size of all endpoints evaluated. Probably due to small sample size, we found only no significant interactions across the studies between J-CTO score ≥ 2 on the incidence of TLR (*p* = 0.21), ISR (*p* = 0.11), ScT (*p* = 0.935). Results of meta-regression analyses are displayed in Additional file 1.

## Discussion

Although the studies leading to their CE marking were mostly based on the analysis of outcomes after treatment of simple lesions, BRS have been used since their introduction in increasingly complex ones. In these settings, including thrombotic, ostial or bifurcation lesions or chronic total occlusions, the potential benefits of vascular resorption could theoretically be larger; on the other side, particularly in light of recent meta-analyses reporting inferior results as compared to modern drug eluting stents in simple lesions [26, 27], this use is not based on evidence and outcomes remain to be reported.

In this study, we summarize the clinical evidence on the use of BRS for the treatment of CTOs. Our reported TLR rate of 3.8% (FU > 11 months) compares favorably with that recently reported by Stone et al. (BRS 2.7%, EES 2.3% at 1 year) in a recent meta-analysis of studies on the use of BRS in simple coronary lesions [28]. As well, the rates of TVR (6.6%), cardiac death (1.1%), scaffold thrombosis (0.9%), clinical scaffold restenosis (1.8%) at mid to long-term follow-up are in line with data reported in previous meta-analyses on the use of DES in CTO lesions. For instance, Yang SS et al. in a meta-analysis of 29 studies [29] reported an incidence of 1.35% of DES thrombosis in this setting at 1-year follow-up, while Colmenarez et al. reported, in another meta-analysis, a TVR rate of 11.71% at 6 to 36 months follow-up [30]. In a recent research letter, Brugaletta et al., suggested the use of ticagrelor in patients undergoing PCI of CTO with the potential to improve vascular function and to reduce TLR and symptoms [31]. Taken together, the present data appear to support the use of BRS in CTO setting.

### Limitations

First, studies with BRS implantation in CTOs are limited in number and mostly single arm, observational and/or include a small sample size. Second, publication bias may have affected the findings of our meta-analysis of published reports. The lack of routine follow-up angiography in most of the studies does not allow detection of the occurrence of some outcomes like restenosis [32]. Third, although we explored the effect of covariates on the effect size, the results of the meta-regression should be carefully interpreted in view of the use of study-level covariates and overall low statistical power [33–35]. Fourth, no data are available on procedural success rates. BRS are bulkier and require a more accurate lesion preparation, which is often harder to achieve in complex lesions [36–41]. Finally, the present data reflect outcomes of BRS in selected centers with expertise in this specific setting, and any assumption of safety should be taken with caution.

## Conclusions

Implantation of BRS in a population with CTO is feasible, although further longer-term outcome studies are necessary.

## Additional file

Additional file 1: Metaregression analyses - The effect of study-level covariates on the rate of TLR, ScR and ScT. (PPTX 161 kb)

## Abbreviations

BRS: Bioresorbable scaffolds
CABG: Coronary artery bypass grafting
CTO: Chronic total occlusions
DES: Drug-eluting stents
MACE: Major adverse cardiovascular events
MI: Myocardial infarction
PCI: Percutaneous coronary interventions
ScR: Scaffold restenosis
ScT: Scaffold thrombosis
TLR: Target-lesion revascularization
TVF: Target vessel failure
TVR: Target-vessel revascularization
