# Supplementary figures and images for: Bioresorbable vascular scaffolds for percutaneous treatment of chronic total coronary occlusions: a meta-analysis
Source: BMC Cardiovasc Disord. 2019 Mar 15;19:59. doi: 10.1186/s12872-019-1042-2 (PMC6419823; doi:10.1186/s12872-019-1042-2)

## Slide 1
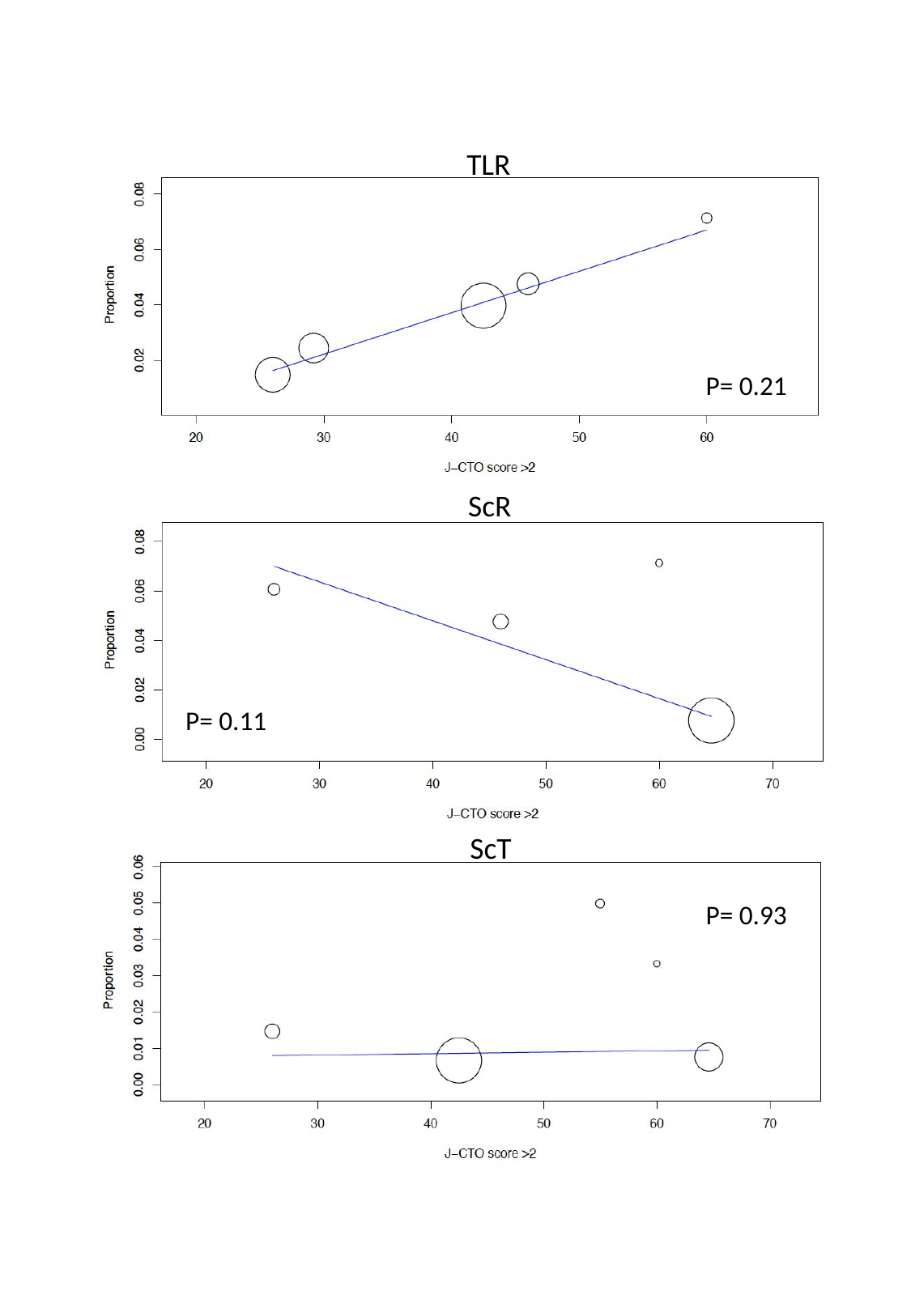

TLR
P= 0.21
ScR
P= 0.11
ScT
P= 0.93

Supplement: Supplementary file 1 — Metaregression analyses - The effect of study-level covariates on the rate of TLR, ScR and ScT. (PPTX 161 kb) [file 12872_2019_1042_MOESM1_ESM.pptx]
